# Hyperreflective Foci in the Outer Retinal Layers as a Predictor of the Functional Efficacy of Ranibizumab for Diabetic Macular Edema

**DOI:** 10.1038/s41598-020-57646-y

**Published:** 2020-01-21

**Authors:** Tatsuya Yoshitake, Tomoaki Murakami, Kiyoshi Suzuma, Yoko Dodo, Masahiro Fujimoto, Akitaka Tsujikawa

**Affiliations:** Department of Ophthalmology and Visual Sciences, Kyoto University Graduate School of Medicine, Kyoto, Japan

**Keywords:** Predictive markers, Outcomes research

## Abstract

Anti-VEGF drugs are as the first-line therapies for diabetic macular edema (DME). In this study, we investigated the association between hyperreflective foci in the outer retinal layers and functional efficacy in DME patients who received intravitreal ranibizumab (IVR) injections. We retrospectively reviewed 77 eyes of 71 patients with DME treated with pro re nata IVR injections for at least 12 months. We evaluated how baseline hyperreflective foci in the outer retinal layers on spectral domain optical coherence tomography images were associated with an improvement in logarithm of the minimum angle of resolution visual acuity (logMAR VA) at 12 months. Forty-three eyes with hyperreflective foci in the outer retinal layers had greater VA improvement than 34 eyes without such foci at 12 months. Multivariate analyses demonstrated that both logMAR VA and hyperreflective foci in the outer retinal layers at baseline were associated with VA improvement. Structural analyses revealed that the central subfield thickness was decreased and that the ellipsoid zone of photoreceptors was improved more significantly in eyes with hyperreflective foci in the outer layers than eyes without such lesions. Baseline hyperreflective foci in the outer retinal layers predict the functional efficacy of IVR injections for DME.

## Introduction

Diabetic macular edema (DME) leads to visual impairment in diabetic patients, mediated via the breakdown of blood-retinal barrier (BRB) and concomitant neuroglial dysfunction^1^. Both basic and clinical researches have shown that vascular endothelial growth factor (VEGF) is a main regulator in the pathogenesis of DME, and anti-VEGF treatment has great effects on anatomical and functional outcomes in DME^2–5^. Two major drugs neutralizing VEGF, ranibizumab and aflibercept, have been used as the first-line therapies for DME in many countries since the approval by the administrations. However, the magnitude of efficacy varies among each patient, indicating a need to find the prognostic factors for anti-VEGF treatment^6,7^.

Spectral domain optical coherence tomography (SD-OCT) has been introduced into the clinical setting and can be used to evaluate the anatomical changes in neuroglial tissues of the retinas^8–13^. Post hoc analyses of a clinical trial found that the presence of subretinal fluid, one of the qualitative OCT findings, predicts better visual outcomes after intravireal ranibizumab (IVR) injections for DME^7^. The qualitative or quantitative parameters in foveal cystoid spaces are also correlated with the short-term anatomical responses to medical treatments^14,15^. Vitreoretinal abnormalities are a predictor of lower efficacy of IVR, whereas epiretinal membrane peeling is associated with greater visual acuity (VA) improvement in eyes that underwent vitrectomy^6,16^. The ellipsoid zone of photoreceptors (EZ) line (or the junction of photoreceptor inner and outer segments[IS/OS]), a marker of foveal photoreceptor status, is restored at 12 months under anti-VEGF treatment, which may partly explain the functional efficacy^17^.

Hyperreflective foci, which appear as highly reflective dots on SD-OCT images, are a sign of vascular hyperpermeability^18^. An initial report hypothesized that these foci may correspond to the precursor of hard exudates or lipid-laden macrophages, and other publications reported that they may also be the debris of photoreceptors or pathological changes in the retinal pigment epithelium (RPE) in the outer layers in chorioretinal diseases^19,20^. Additionally, the amount and distribution of hyperreflective foci or hard exudates change after treatment for DME^21–24^. Hyperreflective foci within the subretinal fluid predict poor visual function after intervention in eyes with serous retinal detachment (SRD), and the foci in the outer retinal layers than the external limiting membrane (ELM) are associated with photoreceptor damage and visual impairment in eyes without SRD^19,25^. Such foci are associated with poor visual outcomes in eyes that receive vitrectomy for DME^26^.

In the current study, we investigated the changes in hyperreflective foci in the outer retinal layers and their association with functional efficacy at 12 months in eyes with center-involved DME treated with pro re nata (PRN) IVR injections^27^.

## Results

### Different visual outcomes between eyes with and without hyperreflective foci in the outer retinal layers

**Figure 1:**
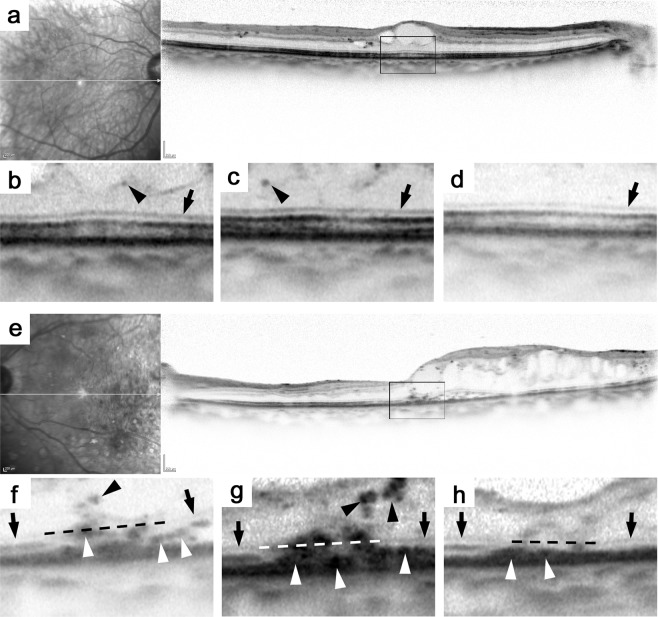
Representative cases treated with PRN IVR injections for DME. (**a–d**) A 74-year-old patient without hyperreflective foci in the outer retinal layers received five IVR injections. The SD-OCT image at baseline (**a**) and the magnified images of the foveal photoreceptors at baseline (**b**), 3 months (**c**), and 12 months (**d**). Despite the resolution of macular edema, logMAR VA worsened from 0.097 to 0.155 at 12 months. (**e–h**) A 73-year-old patient with hyperreflective foci in the outer retinal layers was treated with 4 times of IVR injections. LogMAR VA was improved from 0.699 to 0.222 at 12 months. **(e**) The baseline SD-OCT image dissecting the fovea. Magnified images of the foveal photoreceptors at baseline (**f**), 3 months (**g**), and 12 months (**h**). Black or white arrowheads = hyperreflective foci; black arrow = ELM; dotted line = imaginary line connecting the ELM. (**b,f**) Magnified images of the rectangles in (**a**,**e**).

**Figure 2:**
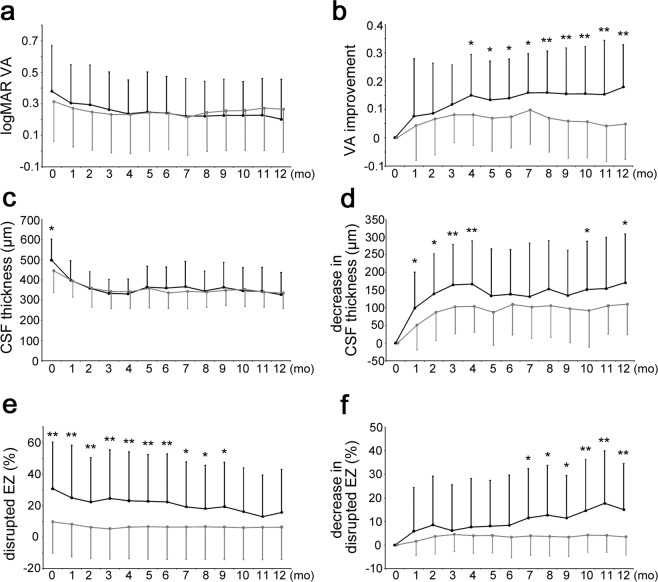
Functional and anatomical outcomes under IVR injections in eyes with and without hyperreflective foci in the outer retinal layers. The course of mean logMAR VA (**a**) and improvement of logMAR VA (**b**). The course of mean CSF thickness (**c**) and its change (**d**). The course of mean disrupted EZ (IS/OS) line (**e**) and its decrease **(f**). Black = 43 eyes with hyperreflective foci in the outer retinal layers; gray = 34 eyes without such foci; error bar = standard deviation. *P < 0.05. **P < 0.01.

After the exclusion of 10 eyes at baseline and 63 eyes that were lost to follow-up before the 12-month visit, we retrospectively reviewed 77 eyes of 71 patients whose characteristics are shown in Table 1. We further presented the patients’ characteristics in cases with and without hyperreflective foci (Tables S1 and S2). Logarithm of the minimum angle of resolution (logMAR) VA improved from 0.352 ± 0.275 to 0.231 ± 0.264 and Central subfield (CSF) thickness decreased from 475 ± 109 μm to 331 ± 96 μm at 12 months under PRN IVR injections. The number of IVR injections was 6.4 ± 2.3 during this period. Hyperreflective foci in the inner or outer retinal layers than the ELM were present at baseline in 64 (83%) or 43 eyes (56%), respectively (Kappa coefficient = 0.828 or 0.838), respectively. Such lesions were decreased to 49 (64%) or 27 eyes (35%), respectively, at 12 months (*P* = 0.010 and *P* = 0.015, respectively; Figs. 1 and 2, Table 2). In particular, 41 eyes (53%) with hyperreflective foci in both layers at baseline were reduced to 21 eyes (27%) at 12 months (Table S3).

**Table 1:** Baseline Characteristics.

Parameter	Value
Eyes/patients	77/71
Age (years) median (IQR)	69 (60–73)
Men/women	39/32
HbA1c (%) median (IQR)	7.1 (6.7–7.8)
Systemic hypertension (patients)	43
LogMAR VA median (IQR)	0.260 (0.155–0.523)
**International classification**	
Mild NPDR	1 eye (1%)
Moderate NPDR	45 eyes (58%)
Severe NPDR	14 eyes (18%)
PDR	17 eyes (22%)
Pseudophakia	27 eyes (35%)
Panretinal photocoagulation	50 eyes (65%)
Hard exudates in the CSF	29 eyes (38%)
CSF thickness (μm) median (IQR)	447 (408–547)
Cystoid abnormalities	64 eyes (83%)
Subretinal fluid	19 eyes (25%)
Disrupted EZ line (%) median (IQR)	9.9 (0.0–29.4)
Hyperreflective foci in the inner retinal layers	64 eyes (83%)
Hyperreflective foci in the outer retinal layers	43 eyes (56%)

**Table 2:** The Course of Hyperreflective Foci in the Inner or Outer Retinal Layers of the ELM under Ranibizumab Injections.

	baseline	1mo	3mo	6mo	9mo	12mo
Hyperreflective foci in the outer retinal layers	43 (56%)	43 (56%)	41 (53%)	42 (55%)	37 (48%)	27 (35%)
Hyperreflective foci in the inner retinal layers	64 (83%)	67 (87%)	60 (78%)	59 (77%)	54 (70%)	49 (64%)

There were no differences in logMAR VA at baseline (0.382 ± 290 vs. 0.315 ± 0.254; *P* = 0.284) or at 12 months (0.202 ± 0.255 vs. 0.266 ± 0.274; *P* = 0.297) between eyes with and without hyperreflective foci in the outer retinal layers (Fig. 2a). The improvement of logMAR VA was greater at 5 months or later in eyes with hyperreflective foci in the outer layers than in eyes without these lesions (0.140 ± 0138 vs. 0.074 ± 0.110; *P* = 0.022 or 0.179 ± 0.150 vs. 0.048 ± 0.124; *P* < 0.001 at 6 or 12 months, respectively; Fig. 2b). In contrast, there were no differences in logMAR VA or VA changes between eyes with and without hyperreflective foci in the inner retinal layers at 12 months. Eyes with hyperreflective foci in the outer retinal layers had VA improvement more frequently than eyes without such foci, in contrast to no differences in the frequency of vision loss (Table 3). The number of IVR injections during 12 months did not differ between eyes with and without hyperreflective foci in the outer retinal layers (Tables S1 and S2).

**Table 3:** Eyes with Vision Gains and Losses in LogMAR VA from Baseline at 12 Months under IVR injections.

Vision Gain, n (%)	Hyperreflective foci in the outer retinal layers (+)	Hyperreflective foci in the outer retinal layers (−)	*P*-value
≥0.3	13 (30%)	1 (3%)	0.002
≥0.2	18 (42%)	3 (9%)	0.002
≥0.1	31 (72%)	12 (35%)	0.002
≥0	38 (88%)	24 (71%)	0.081
Vision Loss, n (%)			
≥0.1	1 (2%)	3 (9%)	0.316
≥0.2	1 (2%)	1 (3%)	1.000

We performed further statistical analyses to find the baseline factors predicting the functional efficacy of IVR injections at 12 months (Table 4). Univariate analyses revealed that preoperative logMAR VA, hard exudates in the CSF (Kappa coefficient = 0.889), the presence of subretinal fluid (Kappa coefficient = 1.000), the transverse length of the disrupted EZ (IS/OS) line (ICC = 0.943), and the presence of hyperreflective foci in the outer retinal layers were associated with improvement of logMAR VA at 12 months, although CSF thickness and cystoid abnormalities (Kappa coefficient = 1.000) were not. We employed the multivariate analysis to adjust for the confounding factors and demonstrated that both logMAR VA and hyperreflective foci in the outer retinal layers at baseline were correlated with VA improvement at 12 months (Table 4).

**Table 4:** Univariate or Multivariate Analyses of Baseline Factors Predicting Improvement in LogMAR VA at 12 Months under Ranibizumab for DME.

Parameters at baseline	univariate			multivariate		
	Unstandardized β	Standardized β	*P*-value	Unstandardized β	Standardized β	*P*-value
Age	−0.001	−0.070	0.544	—	—	—
Gender (male)	−0.010	−0.032	0.784	—	—	—
HbA1c (%)	−0.011	−0.098	0.447	—	—	—
Systemic hypertension	0.039	0.126	0.275	—	—	—
LogMAR VA	0.193	0.347	0.002	0.158	0.284	0.038
PDR	0.014	0.039	0.738	—	—	—
Pseudophakia	−0.030	−0.094	0.416	—	—	—
Panretinal photocoagulation	0.030	0.094	0.414	—	—	—
Hard exudates in the CSF	0.074	0.233	0.042	0.032	0.102	0.347
CSF thickness (μm)	<0.001	0.135	0.243	—	—	—
Cystoid abnormalities	−0.035	−0.086	0.457	—	—	—
Subretinal fluid	0.098	0.279	0.014	0.055	0.156	0.174
Disrupted EZ line (%)	0.002	0.351	0.002	<0.001	0.058	0.697
Hyperreflective foci in the inner retinal layers	0.052	0.129	0.264	—	—	—
Hyperreflective foci in the outer retinal layers	0.131	0.428	<0.001	0.085	0.278	0.028

Univariate logistic regression or Multivariate logistic analysis using significant parameters (P < 0.10) as independent parameters.

### Structural changes in eyes with and without hyperreflective foci in the outer retinal layers

The initial CSF thicknesses were greater in eyes with hyperreflective foci in the outer retinal layers than in eyes without such foci (498 ± 104 μm vs. 445 ± 109 μm; *P* = 0.034), although there were no differences at 1month or later time points (Fig. 2c). The decrease in CSF thickness was more significant in eyes with such foci than in eyes without them at 12 months (171 ± 138 μm vs. 110 ± 86 μm; *P* = 0.028; Fig. 2d). Recent publications reported an association between photoreceptor damage and hyperreflective foci in the outer retinal layers, which prompted us to investigate the changes in the EZ (IS/OS) line^19^. The transverse length of the disrupted EZ (IS/OS) line was longer in eyes with such foci at baseline (30.8 ± 29.8% vs. 9.8 ± 20.0%; *P* < 0.001), whereas the length did not differ between eyes with and without these lesions at 12 months (15.7 ± 27.2% vs. 7.5 ± 21.3%; *P* = 0.154; Fig. 2e). The disrupted EZ (IS/OS) line was shortened more greatly in eyes with hyperreflective foci in the outer retinal layers than in those without such foci at 12 months (15.0 ± 19.5% vs. 2.2 ± 9.6%; *P* < 0.001; Fig. 2f).

## Discussion

Anti-VEGF treatment is the first-line strategy for DME worldwide, although other interventions, e.g., photocoagulation, steroids, and vitrectomy, may sometimes be applied or combined with anti-VEGF agents^4,28,29^. This may prompt ophthalmologists to consider the importance of prognostic factors for individual interventions to progress toward personalized medicine. However, only a few publications have reported the prognostic factors under anti-VEGF therapy for DME^6,7^. Since hyperreflective foci represent extravasation on SD-OCT images, we in this study investigated whether this OCT finding predicts visual outcomes under PRN IVR injections for DME^18^. Multivariate analysis demonstrated that, among several systemic and ocular parameters, including OCT findings at baseline, both logMAR VA and hyperreflective foci in the outer retinal layers were correlated with VA improvement at 12 months. This suggests that such foci are a novel imaging biomarker predicting the functional efficacy of IVR treatment for DME.

Preoperative hyperreflective foci in the outer retinal layers were related to VA improvement at 12 months under IVR injections for DME. Structural analyses using OCT images revealed that such foci also predicted a greater decrease in CSF thickness at 12 months. CSF thickness is a main surrogate marker during DME management, and these functional and structural efficacies of anti-VEGF treatment are consistent with each other^8,30^. A few publications have reported that hyperreflective foci in the outer retinal layers are related to a well-known biomarker of visual impairment, foveal photoreceptor damage^19,23,25,26^. This encouraged us to investigate the changes in the foveal EZ (IS/OS) lines during IVR treatment. Eyes with hyperreflective foci in the outer retinal layers had a greater disruption in EZ (IS/OS) lines at baseline and their greater improvement at 12 months than eyes without such foci. A recent publication demonstrated the association between VA improvement and a decrease in disrupted EZ (IS/OS) lines after anti-VEGF treatment for DME^17^. This allows us to hypothesize that greater VA improvement can be partly explained by foveal photoreceptor restoration in eyes with hyperreflective foci in the outer retinal layers.

Hyperreflective foci in the outer retinal layers might correspond to deposited lipoproteins, lipid-laden macrophages, debris of photoreceptors, and RPE hyperplasia^18–20^. The former publication discussed that hyperreflective foci may correspond to deposited lipoproteins or lipid-laden macrophages, which represent extravasation and neuroinflammation. Hyperreflective foci in the inner and outer layers were reduced at later time points. This decrease may be explained by the blockade of vascular hyperpermeability to some extent^2^. However, photoreceptor damage and retinal thickening were restored in both early and late time points. Anti-VEGF treatment thus might inhibit VEGFR1-dependent activation of microglia/macrophages and concomitantly recover structural damage regardless of the presence of these inflammatory cells^31^. Another possibility might be that eyes with hyperreflective foci in the outer retinal layers had reversible and half-broken photoreceptors, which were restored under reduced hyperpermeability by anti-VEGF treatment.

Hyperreflective foci in both the inner and outer retinal layers were decreased at the later time points under PRN IVR injections in this study. This result is consistent with the decreased number of hyperreflective foci or spots or the decreased cases with hard exudates after anti-VEGF treatment found in previous studies^23,24,32^. Especially, Kang and associates declared that hyperreflective foci predict poor visual outcomes after PRN intravitreal bevacizumab (IVB) injections^23^. In the current study, logMAR VA did not differ at 12 months between eyes with and without hyperreflective foci in the outer retinal layers, whereas eyes with such foci had greater VA improvement. This discrepancy might be partly explained by the different durations of follow-up, the different drugs, and the different methods used to assess hyperreflective foci. Additionally, hyperreflective foci in the inner retinal layers were not associated with VA improvement in this study, in contrast to those in the outer layers. Signal transduction from foveal photoreceptors is mediated via bipolar cells and ganglion cells in the inner layers of the extrafoveal areas rather than of the fovea. Consequently, future studies should elucidate how hyperreflective foci in the extramacular inner layers can predict the efficacy of IVR injections for DME. Another explanation might be the different macrophage polarization between inner and outer retinal layers^33^. Hyperreflective foci in the inner retinal layers might correspond to anti-inflammatory macrophages and rescue neuroretinas in a VEGF-independent manner. Hyperreflective foci in the outer retinal layers, as proinflammatory macrophages, might exacerbate neuroinflammation, which is reversed by anti-VEGF drugs.

Several publications advocated foveal morphologies as predictors of functional outcomes after anti-VEGF treatment for DME^7,15,34–36^. In particular, the post hoc analysis of the RISE/RIDE studies demonstrated that the presence of subretinal fluid predicts better visual gain after two years of monthly IVR injections^7^. This result agrees with the greater VA improvement in eyes with subretinal fluid found in the univariate analysis of this study. Seventeen eyes (40%) with hyperreflective foci in the outer retinal layers had subretinal fluid, whereas only 2 eyes (6%) without such foci were accompanied by SRD. In addition, there was an association between the disrupted EZ (IS/OS) line and hyperreflective foci in the outer retinal layers, and several publications reported photoreceptor damage as a predictor of poor visual outcomes^19,25,35,37^. Significantly, multivariate analysis designated subretinal fluid and the disrupted EZ (IS/OS) line as confounders of hyperreflective foci in the outer retinal layers. Thus, hyperreflective foci in the outer retinal layers represent vascular hyperpermeability and can be an upstream regulator of these structural changes in neuroretinas.

Previous publications have suggested that the treatment frequency and duration of DME are also associated with functional efficacy under anti-VEGF treatment. The frequent injections of expensive anti-VEGF drugs are a socioeconomic burden. The comparisons of visual outcomes between several clinical trials have suggested that VA improvement is positively correlated to the treatment frequency^38,39^. The number of IVR injections during 12 months did not differ between eyes with and without hyperreflective foci in the outer retinal layers. It suggests that this OCT finding is feasible to optimize the indication of anti-VEGF treatment, independent of socioeconomic concerns. The other predictor is the duration of DME or symptoms, as shown in several publications^39^. Since most patients were referred to our institutes, we could neither know the exact onset of DME nor investigate the relevance of this parameter. Future study should elucidate the relationship between the duration of symptoms and hyperreflective foci in the outer retinal layers.

This retrospective study with a small number of cases had several potential limitations. All participants were from a single center, Asian and treated according to PRN dosing for only 12 months. Additional multi-center studies should confirm the reproducibility in other races managed using other regimens after longer follow-up^40,41^. Several OCT parameters were subjectively evaluated, and further objective and automated detection of retinal lesions should be developed to improve the reproducibility. Ultimately, future comparative studies to other interventions would help guide us to personalized medicine.

In conclusion, we demonstrated that, among several OCT parameters, preoperative hyperreflective foci in the outer retinal layers are a novel predictor of VA improvement at 12 months under PRN IVR injections for DME.

## Methods

### Participants

In this retrospective study, we reviewed 77 consecutive eyes of 71 patients who had a baseline visit at Kyoto University Hospital from March 2014 to February 2017 as the baseline visit and received PRN IVR injections for DME. The inclusion criterion was center-involved DME that was treated with IVR administration according to the 3 + PRN regimen for at least 12 months. The exclusion criteria were eyes with media opacity leading to visual reduction, other chorioretinal diseases, treatment for DME within 6 months, previous intraocular surgery other than cataract surgery, or cataract surgery within 3 months. We additionally excluded the cases which dropped out during 12-months follow-up due to a patient’s desire to terminate treatment or switch to other treatment, patients’ inconvenience, drug tachyphylaxis, or additional treatments, i.e., panretinal photocoagulation, cataract surgery, vitreoretinal surgery (for vitreous hemorrhage), or focal/grid photocoagulation.

All research and measurements adhered to the tenets of the Declaration of Helsinki after the approval of the study protocol by the Kyoto University Graduate School and Faculty of Medicine, Ethics Committee. All participants provided written informed consent before study enrollment.

### Intervention

In this study, IVR administration was performed according to the 3 + PRN regimen described in the Ranibizumab Monotherapy or Combined with Laser versus Laser Monotherapy for Diabetic Macular Edema (RESTORE) study^38^. Ranibizumab (0.5 mg) was injected 3.5 mm posterior to the limbus after disinfection. After three monthly loading doses, PRN IVR injections were considered at monthly visits according to the retreatment criteria of the RESTORE study, which is the standard-of-care therapy for DME.

### Fundus imaging

The best-corrected decimal VA was measured and converted to logMAR VA at every monthly visit. After the comprehensive ophthalmic examinations, we obtained retinal sectional images using SD-OCT (Spectralis OCT, Heidelberg Engineering, Heidelberg, Germany). Vertical and horizontal retinal sectional images dissecting the fovea were acquired using the 30-degree cross-hair mode, and 20 to 100 images were averaged to create better images as previously described^42^. Three-dimensional images were obtained using the raster scan mode, and two-dimensional maps were constructed, which enabled us to measure the CSF thickness of the Early Treatment Diabetic Retinopathy Study (ETDRS) grid (the mean retinal thickness within a 1 mm circle centering on the fovea), as previously described^42^.

We qualitatively and quantitatively evaluated several parameters within the central 1 mm in both vertical and horizontal sections. Hyperreflective foci between the ELM and the surface of the RPE were defined as those in the outer retinal layers, as previously described^19,26^. When the ELM was disrupted at the fovea, we drew an imaginary straight line connecting the ELM line in the parafovea and determined the presence or absence of hyperreflective foci in the outer layers (Fig. 1f). The foveal photoreceptor status was manually evaluated according to a previous publication^43^. After the status of the EZ line was classified into three categories, i.e., disrupted, faint, and intact, we measured the transverse length of the disrupted EZ (IS/OS) line within the central 1 mm on both vertical and horizontal sectional images, and the average was calculated. We further assessed three qualitative findings, i.e., cystoid abnormalities, and subretinal fluid, as previously described^6^. Two retinal specialists evaluated these OCT parameters. Disagreements were discussed until a consensus was reached regarding qualitative OCT findings, and the average of the quantitative parameters was applied for further analyses.

Hard exudates in the CSF were evaluated on color fundus photographs according to the modified methods described previously^24^. After mydriasis, we obtained 40-degree fundus photographs centering on the fovea using a color fundus camera (TRC-NW6S, Topcon, Tokyo, Japan). Two retinal specialists assessed the severity of hard exudates based on the standard photographs on a diagnostic monitor (FlexScan SX2762W, EIZO Co., Ishikawa, Japan)^44^. The central circle (nominal 1 mm diameter = 2/3 disk diameter) was defined as the CSF on fundus photographs^24^. ‘Definite’ or more severe grades of hard exudates in the CSF were categorized into ‘hard exudates in the CSF’.

### Statistics

The results are expressed as the mean ± standard deviation. After test of normal distribution, the Student’s t-test or Mann-Whitney U-test was used for the parametric or non-parametric datasets. Fisher’s exact test or chi-square test was applied to evaluate the sampling distribution. The Kappa coefficient or intraclass correlation coefficient (ICC) was employed to assess the concordance in the qualitative or quantitative parameters, respectively. Linear regression analyses were applied to evaluate whether baseline characteristics (independent variables; age, gender, HbA1c, systemic hypertension, logMAR VA, proliferative diabetic retinopathy (PDR), pseudophakia, panretinal photocoagulation, hard exudates in the CSF, CSF thickness, cystoid abnormalities, subretinal fluid, disrupted EZ (IS/OS) line, hyperreflective foci in the inner retinal layers, and those in the outer retinal layers) were associated with the improvement of logMAR VA at 12 months. Significant factors in univariate analyses (*P* < 0.10) were applied for multiple linear regression analysis. We used SPSS version 24.0 for statistical analyses (SPSS, Inc., Chicago, IL, USA).

## Supplementary information

Supplementary information.
