# Supplementary material for: Hyperreflective Foci in the Outer Retinal Layers as a Predictor of the Functional Efficacy of Ranibizumab for Diabetic Macular Edema
Source: Sci Rep. 2020 Jan 21;10:873. doi: 10.1038/s41598-020-57646-y (PMC6972781; doi:10.1038/s41598-020-57646-y)
Supplement: Supplementary file 1 — Supplementary information. [file 41598_2020_57646_MOESM1_ESM.pdf]

Supplementary Information

Hyperreflective Foci in the Outer Retinal Layers as a Predictor of the Functional Efficacy of Ranibizumab for Diabetic Macular Edema

Tatsuya Yoshitake, MD, Tomoaki Murakami, MD, PhD, Kiyoshi Suzuma, MD, PhD, Yoko Dodo, MD, PhD, Masahiro Fujimoto, MD, PhD, Akitaka Tsujikawa MD, PhD.

From the Department of Ophthalmology and Visual Sciences, Kyoto University Graduate School of Medicine, Kyoto, Japan.

Correspondence and requests for materials should be addressed to Tomoaki Murakami, MD, Department of Ophthalmology and Visual Sciences, Kyoto University Graduate School of Medicine, 54 Shogoin-Kawaracho, Sakyo, Kyoto 606-8507, Japan (phone: 81-75-751-3250; fax: 81-75-752-0933; email: mutomo@kuhp.kyoto-u.ac.jp)

E-mail: mutomo@kuhp.kyoto-u.ac.jp.

Table S1. Patients' characteristics in cases with and without hyperreflective foci in the inner retinal layers.

Parameter	Hyperreflective foci in the inner retinal layers		*P*-value
	absent	present	
Eyes/patients	13/13	64/58	-
Age (years) median (IQR)	70 (65-74)	69 (60-73)	0.429
Men/women	5/8	34/24	0.227
HbA1c (%) median (IQR)	7.2 (6.7-7.6)	7.1 (6.7-7.9)	0.876
Systemic hypertension (patients)	8	35	1.000
LogMAR VA median (IQR)	0.301 (0.155-0.602)	0.260 (0.155-0.473)	0.255
International classification			0.611
Mild NPDR	0 eye	1 eye	
Moderate NPDR	8 eyes	37 eyes	
Severe NPDR	1 eye	13 eyes	
PDR	4 eyes	13 eyes	
Pseudophakia	5 eyes	22 eyes	0.761
Panretinal photocoagulation	9 eyes	41 eyes	1.000
Hard exudates in the CSF	1 eye	28 eyes	0.025
CSF thickness (μm) median (IQR)	408 (318-515)	455 (410-550)	0.086
Cystoid abnormalities	12 eyes	52 eyes	0.449
Subretinal fluid	1 eye	18 eyes	0.167
Disrupted EZ line (%) median (IQR)	0.0 (0.0-9.3)	14.3 (0.0-33.8)	0.696
Hyperreflective foci in the outer retinal layers	2 eyes	41 eyes	0.002
The number of IVR injections	6 (5-7)	7 (4-8)	0.374

Table S2. Patients' characteristics in cases with and without hyperreflective foci in the outer retinal layers.

Parameter	Hyperreflective foci in the outer retinal layers		*P*-value
	Absent	present	
Eyes/patients	34/33	43/38	-
Age (years) median (IQR)	69 (66-74)	68 (58-73)	0.229
Men/women	18/15	21/17	1.000
HbA1c (%) median (IQR)	7.5 (6.8-8.1)	7.0 (6.3-7.5)	0.222
Systemic hypertension (patients)	19	24	0.808
LogMAR VA median (IQR)	0.222 (0.155-0.456)	0.301 (0.155-0.523)	0.292
International classification			0.653
Mild NPDR	1 eye	0 eye	
Moderate NPDR	21 eyes	24 eyes	
Severe NPDR	5 eyes	9 eyes	
PDR	7 eyes	10 eyes	
Pseudophakia	12 eyes	15 eyes	1.000
Panretinal photocoagulation	20 eyes	30 eyes	0.346
Hard exudates in the CSF	6 eyes	23 eyes	0.002
CSF thickness (μm) median (IQR)	417 (389-505)	478 (425-564)	0.034
Cystoid abnormalities	32 eyes	32 eyes	0.031
Subretinal fluid	2 eyes	17 eyes	0.001
Disrupted EZ line (%) median (IQR)	0.0 (0.0-10.2)	20.1 (6.0-52.0)	<0.001
Hyperreflective foci in the inner retinal layers	23 eyes	41 eyes	0.002
The number of IVR injections	6 (4-7)	7 (4-9)	0.097

Table S3. The Course of Hyperreflective Foci in the Inner or Outer Retinal Layers of the ELM under Ranibizumab Injections.

	baseline	1mo	3mo	6mo	9mo	12mo
No hyperreflective foci	11 (14%)	9 (12%)	14 (18%)	14 (18%)	15 (19%)	22 (29%)
Hyperreflective foci in the outer retinal layers alone	2 (3%)	1 (1%)	3 (4%)	4 (5%)	8 (10%)	6 (8%)
Hyperreflective foci in the inner retinal layers alone	23 (30%)	25 (32%)	22 (29%)	21 (27%)	25 (32%)	28 (36%)
Hyperreflective foci in both the inner and outer retinal layers	41 (53%)	42 (55%)	38 (49%)	38 (49%)	29 (38%)	21 (27%)
